# Relationship between clinical examination findings and objective nasal patency measures in structural nasal obstruction: a systematic review

**DOI:** 10.1017/S002221512400210X

**Published:** 2025-05

**Authors:** Pavithran Maniam, Ian Wei Lim, Kim Hui Lim, Sean Carrie, James O’Hara

**Affiliations:** 1 Ear, Nose & Throat Department, Newcastle upon Tyne Hospitals NHS Foundation Trust, Newcastle upon Tyne, UK; 2 The University of Sheffield Medical School, Sheffield, UK; 3 Ear, Nose & Throat Department, Northwest Anglia NHS Foundation Trust, Peterborough, UK; 4 Honorary Affiliation with Translational and Clinical Research Institute, Newcastle University, Newcastle upon Tyne, UK; 5 Population Health Sciences Institute, Newcastle University, Newcastle upon Tyne, UK

**Keywords:** anatomy, diagnosis, nasal physiology, rhinology, rhinomanometry, rhinometry, acoustic, systematic reviews

## Abstract

**Background:**

The relationship between clinical examination findings and objective nasal patency measures in structural nasal obstruction remains uncertain. This review aims to explore the relationship between clinical nasal examination findings and objective nasal patency measures using acoustic rhinometry, peak nasal inspiratory flow, rhinomanometry and rhinospirometry.

**Methods:**

Qualitative systematic review using the Preferred Reporting Items for Systematic reviews and Meta-Analyses 2020 statement.

**Results:**

A total of 17 articles were included in the systematic review. Several studies showed a positive relationship between objective nasal patency measures and clinical nasal examination findings, however evidence in the literature is limited and confined to cohort studies. Objective nasal patency measures using acoustic rhinometry, rhinomanometry and rhinospirometry assessment correlate positively in severe anterior septal deviation but its role in assessing middle/posterior and mild/moderate septal deviation in isolation remains uncertain. There is limited evidence in the literature to assess the relationship between peak nasal inspiratory flow and clinical examination findings.

**Conclusion:**

Objective nasal patency measures has a limited role in supporting clinical examination findings in severe structural nasal obstruction.

## Introduction

Nasal obstruction can be caused by structural nasal deformities or be due to inflammatory processes affecting the nasal mucosa.^1^ Structural causes of nasal obstruction include deviation of septal cartilage and/or the nasal bones, turbinate enlargement or deformities of the alar cartilage.^1^ Septal deviation is a common cause of chronic upper airway nasal obstruction and septoplasty is commonly performed to relieve symptomatic nasal obstruction secondary to septal deviation.^2^ In the National Health Service (NHS) in England, approximately 17,000 septoplasties are performed annually,^2^ while in the United States, approximately 250,000 septoplasties are performed annually.^3^

Septoplasty provides functional and objective benefits for patients with chronic nasal obstruction secondary to structural deformities by improving nasal airflow.^4^^–^^6^ However, patient selection for septoplasty remains undefined. The diagnosis of nasal obstruction is predominantly based on clinical history from patients and clinical examination of the nasal valve, lateral nasal wall, septum and inferior turbinate.^7^ Subjective patient-reported symptom scoring tools such as the Nasal Obstruction Symptom Evaluation (NOSE)^8^ and Sino-Nasal Outcome Test (SNOT-20) scores^9^ are available but used infrequently in clinical settings to assess the severity of nasal obstruction.

Objective nasal patency measuring tools such as acoustic rhinometry, rhinomanometry, rhinospirometry and peak nasal inspiratory flow (PNIF) are also available to assess the degree of nasal obstruction. Acoustic rhinometry measures the cross-sectional areas and nasal volumes using reflected sound waves from the nasal cavities to establish the degree of nasal patency.^10^ Valleys in acoustic rhinometry graphs represent reductions in cross-sectional area at specific distances from the interface and these valleys represent a defined anatomic structure.^11^ Cross-sectional areas (CSAs) are dips on acoustic rhinometry. CSA1 corresponds to the area of the nasal valve, CSA 2 to the inferior and/or medial nasal concha and CSA3 corresponds to the medial-posterior end of the medial nasal concha. Rhinomanometry measures trans-nasal airflow and pressure to establish nasal airway resistance during inspiration.^12^ Rhinospirometry measures the difference in volume, average flow, peak flow and partitioning of airflow between the nasal passages.^13^ The reference pressures of 75 and 150 Pascal (Pa) are often used to calculate the expiratory and inspiratory nasal airway resistances. In rhinospirometry, the nasal partitioning ratio is a measure of asymmetry of airflow through the nasal cavities that ranges from -1 (complete left nostril obstruction) to +1 (complete right nostril obstruction), with 0 indicating symmetrical airflow.^14^ PNIF measures maximal airflow during forced nasal inspiration through both nostrils.^12^

Despite evidence demonstrating objective improvement in measures of nasal patency and airflow following septoplasty,^4^ the clinical applicability of objective nasal patency measures using acoustic rhinometry, PNIF, rhinomanometry and rhinospirometry for selecting patients with structural nasal obstruction for septoplasty remains undefined.

This systematic review aims to explore the currently available evidence on the relationship between clinical nasal examination findings in structural nasal obstructions and objective nasal patency measures using acoustic rhinometry, PNIF, rhinomanometry and rhinospirometry. This will allow clinicians to decide if objective nasal patency measures can be used alongside clinical examination findings to aid patient selection for septoplasty.

## Methods

### Ethical consideration

No patient-identifiable data are included. This study is a systematic review of previously published articles. No ethical approval was required.

### Data search strategy

**Figure 1.**
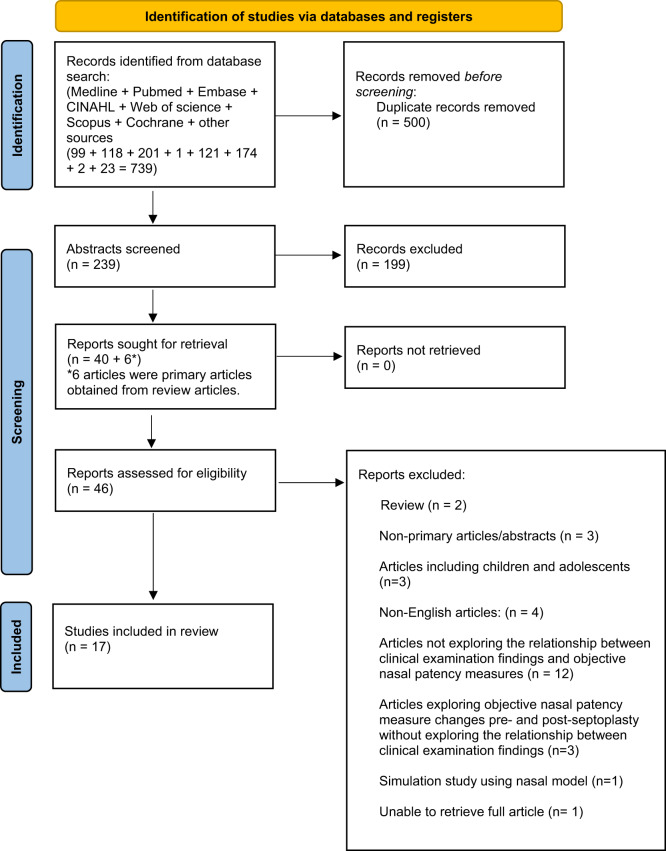
Workflow of article screening for final narrative synthesis.

This systematic review was registered with the International Prospective Register of Systematic Reviews (PROSPERO) (Registration number: CRD 42023417330).^15^ A systematic literature search was performed using Medical Subject Heading (MeSH) terms and other keywords as outlined by the Preferred Reporting Items for Systematic Reviews and Meta‐Analyses (PRISMA) 2020 statement.^16^ Pubmed (US National Library of Medicine), Medline, Embase, SCOPUS, Web of Science, Cumulated Index to Nursing and Allied Health Literature (CINAHL), Cochrane Library and Google Scholar databases were used. The final literature search was performed on 17/05/2024. The MeSH terms and search strategy for this systematic review are provided in Appendix 1. The inclusion and exclusion criteria are outlined in Table 1. The Oxford Centre for Evidence-Based Medicine guidelines were used to establish the level of evidence of scientific articles.^17^ The workflow for the systematic review is outlined in Figure 1.

**Table 1.** Inclusion and exclusion criteria for systematic review

Criteria	Inclusion	Exclusion
Study description	Articles that explored the relationship between clinical examination (using video endoscope, nasal endoscope or anterior rhinoscope) findings with objective nasal patency measure using either PNIF, acoustic rhinometry, rhinospirometry or rhinomanometry.	Articles that did not explore the relationship between clinical examination (using video endoscope, nasal endoscope or anterior rhinoscope) findings with objective nasal patency measure using either PNIF, acoustic rhinometry, rhinospirometry or rhinomanometry.
Article type	All primary articles	Secondary articles such as review articles and abstracts
Age	Adults above 18 years old	Adolescents and children (age less than 18 years old)
Language	English language articles	Non-English language articles
Time period	No restriction on publication date	Not applicable

Following data synthesis, findings from the literature are summarised according to the type of objective nasal patency measures. This allows comparison of evidence from the literature based on the type of objective nasal patency measure and to establish how individual objective measures correlate with clinical examination findings. The strength of correlation reported in each study is classified as described by Hensch and Evans^18^ as “very weak correlation” (correlation coefficient of 0.00–0.19), “weak correlation” (0.20–0.39), “moderate correlation” (0.40–0.59), “strong correlation” (0.60–0.79) and “very strong correlation” (0.80–1.0). Cohen k statistics were also used to describe the agreement between the different outcome measures. A kappa score of less than 0 indicates no agreement, 0.01–0.20 slight agreement, 0.21–0.40 fair agreement, 0.41–0.60 moderate agreement, 0.61–0.80 substantial agreement and 0.81–0.99 almost perfect agreement.^19^

## Results

A total of 17 articles met the inclusion criteria (Table 1) and were included in the review (Tables 2–5).

**Table 2.** Studies investigating the correlation between clinical examination findings and acoustic rhinometry

Study	Country	Evidence type/level	No. of participants (n)	Objective nasal patency measure	Summary of findings
Ouriques *et al*. (2006)^20^	Canada	Cohort study/ level 4	67	Acoustic rhinometry	Moderate to strong correlation between first three constrictions detected by acoustic rhinometry and first three anatomic narrowing (i.e., nasal valve, inferior nasal turbinate and head of middle turbinate respectively) on nasal endoscopy. The correlation coefficient between acoustic rhinometry measurement and anatomical nasal narrowing at the nasal valve, head of the inferior turbinate and head of the middle turbinate was +0.442, +0.763 and +0.728, respectively. Very weak correlation between the fourth constriction on acoustic rhinometry with choanal region (correlation co-efficient: +0.081).
Corey *et al*. (1999)^11^	USA	Cohort study/level 4	85	Acoustic rhinometry	The first, second and third acoustic rhinometry valley correlated to the nasal valve, inferior turbinate and middle turbinate respectively. Valleys identified on acoustic rhinometry did not provide exact point to point correlation with nasal cavity examination.
Trindade *et al*. (2013)^21^	Brazil	Cohort study/level 4	30	Acoustic rhinometry	The minimal cross-sectional values (i.e., CSA1, CSA 2 and CSA 3) and nasal volumes were lower in patients with nasal obstruction secondary to septal deviation with or without turbinate hypertrophy when compared to control in acoustic rhinometry. The distance from the nostril to the point of cross-sectional values (dCSA 1 and dCSA2) was higher in the nasal obstruction group.
Huang *et al*. (2009)^23^	Singapore	Cohort study/level 4	189	Acoustic rhinometry	Individuals with inferior turbinate hypertrophy had lower minimal cross-sectional values and total nasal volume when compared to individuals with rhinoscopically normal nose. The minimal cross-sectional values and total nasal volume were also lower in individuals with septal deviation when compared to individuals with inferior turbinate hypertrophy.
Tantilipikorn *et al*. (2008)^24^	Thailand	Cohort study/level 4	135	Acoustic rhinometry	No significant difference in mean minimal cross-sectional area, distance from the nostril to the point of cross-sectional values and nasal volume between patients with mild septal deviation and without septal deviation pre-nasal decongestant. A difference in minimal cross-sectional area and nasal volume was evident postnasal decongestant.
Pirila *et al*. (2009)^25^	Finland	Cohort study/level 4	110	Acoustic rhinometry and rhinomanometry	Post-decongestant overall minimal cross-sectional area on the side of septal deviation predicted post-operative satisfaction (*p* < 0.01). The optimal suggested cutoff value of the overall minimal cross-sectional area was 0.40 cm^2^. The sensitivity and specifity of the cut of value were 65 per cent and 60 per cent for patients reporting high or very high post-operative satisfaction. In severe septal deviation, anterior rhinoscopy is predictive of post-operative satisfaction but for milder septal deviation, acoustic rhinometry was better at predicting post-operative success when compared to anterior rhinoscopy.
Scüzs *et al*. (1998)^22^	Belgium	Cohort study/level 4	50	Acoustic rhinometry and rhinomanometry	Minimal cross-sectional area was predictive of severe deviations in the anterior nasal cavity but less predictive for middle or posterior deviation.

### Relationship between clinical examination findings and acoustic rhinometry

Studies exploring the relationship between clinical examination findings and acoustic rhinometry were level 4 cohort studies/case series (Table 2). Positive relationships between clinical examination findings and acoustic rhinometry assessment were observed in some studies^11^^,^^20^^–^^23^ (Table 2). Ouriques *et al*. compared and correlated areas of anatomical nasal narrowing on fibreoptic nasal endoscopy with acoustic rhinometry measurements in individuals without nasal complaints. A moderate to strong correlation was identified among the first three constrictions detected on acoustic rhinomanometry and the first three parts of anatomic narrowing (i.e., nasal valve, head of the inferior turbinate and head of the middle turbinate) on nasal endoscopy. A very weak correlation was evident between the fourth constriction measurement on acoustic rhinometry and the fourth anatomic constriction which is the posterior choanal region^20^ (Table 2). Similarly, Corey *et al*. highlighted that the first valley on acoustic rhinometry correlated with the nasal valve anatomy, the second valley corresponded to the anterior aspect of the inferior turbinate and the third valley correlated to the anterior aspect of the middle turbinate. However, these valley measurements on acoustic rhinometry failed to provide an exact point-to-point correlation with the nasal cavity on clinical examination (Table 2).^11^

**Table 3.** Studies investigating the correlation between clinical examination findings and rhinomanometry

Study	Country	Evidence type/level	No. of participants (n)	Objective nasal patency measure	Summary of findings
Scüzs *et al*. (1998)^22^	Belgium	Cohort study/level 4	50	Rhinomanometry	Anterior nasal volume, inspiratory and expiratory nasal airway resistance (NAR) at 75 Pa and at 150 Pa were predictive of severe deviations in the anterior nasal cavity but less predictive for middle or posterior deviation (*p* < 0.05).
Panagou *et al*. (2009)^26^	Greece	Cohort study/level 4	254	Modified rhinomanometry	Moderate correlation between modified rhinomanometry (i.e., occlusion method) and clinical assessment (r = 0.57, *p* = 10^−^^4^) of nasal airflow on a six-point scale.
Bock *et al*. (2017)^27^	Germany	Cohort study/level 4	124	Rhinomanometry	Rhinomanometric total inspiratory flow and rhinoscopy findings were not significantly associated in patients with cystic fibrosis.
Tompos *et al*. (2010)^28^	Hungary	Cohort study/level 4	86	Rhinomanometry	Very weak correlation between rhinoscopy findings and nasal airflow resistance on rhinomanometry at 75 and 150 Pa.
Huygen *et al*. (1992)^29^	Netherlands	Cohort study/level 4	193	Rhinomanometry	Abnormally low flow rates on rhinomanometry were detected in patients with no septal deviation or mild deviation restricted to one anatomical area. The mean detection rates of small, moderate and large septal deviations were 2 per cent, 36 per cent and 76 per cent, respectively on rhinomanometry.
McCaffrey *et al*. (1979)^30^	USA	Cohort study/level 4	1000	Rhinomanometry	Patients with abnormalities on rhinoscopic examination had near normal nasal resistances. Rhinoscopic evidence of nasal deformity does not predict increase in nasal resistance.
Pirila *et al*. (2009)^25^	Finland	Cohort study/level 4	110	Rhinomanometry	Post-decongestant inter-cavital airflow on the side of septal deviation predicted post-operative satisfaction. The optimum suggested cutoff value of inter-cavital airflow is 1:2. The sensitivity and specificity of this cutoff value were 65 per cent and 60 per cent for patients reporting high or very high post-operative satisfaction. In severe septal deviation, anterior rhinoscopy is predictive of post-operative satisfaction but for milder septal deviation, rhinomanometry were better at predicting post-operative success when compared to anterior rhinoscopy.
Sipilla *et al*. (1997)^31^	Finland	Cohort study/level 4	432	Rhinomanometry	Patients with high pre-operative intranasal resistance on rhinomanometry had a higher post-operative satisfaction level when compared to patients with normal resistance.
Suonpää *et al*. (1993)^32^	Finland	Cohort study/level 4	102	Rhinomanometry	Patients with high post-operative satisfaction had high pre-operative nasal airway resistance on rhinomanometry. Patients with airway resistance within normal limits remained symptomatic and were less satisfied post-operatively.

Other studies also reported findings whereby the minimal cross-sectional area values and nasal volumes were lower in patients with nasal obstruction when compared to healthy controls suggesting that most patients with structural nasal obstruction had impairment in acoustic rhinometry assessment due to anatomical differences^21^^–^^23^ (Table 2). Trindade *et al*. highlighted that the minimal cross-sectional values were lower in patients with septal deviation when compared to healthy control whereas the distance from the nostril to the point of cross-sectional values was higher.^21^ Scüzs *et al*. also reported that the minimal cross-sectional area is lower in patients with septal deviation when compared to the control cohort although the minimal cross-sectional area was more sensitive in identifying severe deviations in the anterior nasal cavity when compared to the middle or posterior septal deviation.^22^

A study by Tantilipikorn *et al*. explored the relationship between acoustic rhinometry measurements and clinical examination findings using anterior rhinoscopy in asymptomatic adults with mild septal deviation or without septal deviation. In this study, no difference in pre-decongestant minimal cross-sectional area, nasal volume and distance from nostril to the point of cross-sectional values were observed between patients with mild septal deviation and patients without septal deviation. However, a difference in post-decongestant minimal cross-sectional area and nasal volume was observed in these groups of patients^24^ (Table 2).

Pirila *et al*. assessed the degree of septal deviation using anterior rhinoscopy and classified deviations as “very severe” if total or sub-total obstruction was noted, “severe” if more than 50 per cent obstruction was noted and “moderate” or “mild” if less than 50 per cent of the airway was obstructed by the deviation. Post-operative satisfaction was expressed by patients as “very high”, “high”, “moderate”, or “low” one year after septoplasty. Post-decongestion overall minimal cross-sectional area of the deviated septum on acoustic rhinometry performed pre-operatively had the highest impact in identifying patients with high or very high post-operative satisfaction. With anterior rhinoscopy, the optimum cutoff value to achieve high post-operative satisfaction is between “severe” and “moderate” septal deviation. Pirila *et al*. concluded that, for patients with very severe septal deviation, anterior rhinoscopy alone was sufficient to screen for post-operative satisfaction but for patients with milder septal deviation, acoustic rhinometry was a better screening tool to assess post-operative satisfaction when compared to anterior rhinoscopy^25^ (Table 2).

### Relationship between clinical examination findings and rhinomanometry

Studies exploring the relationship between clinical examination findings and rhinomanometry are shown in Table 3. Scüzs *et al*. highlighted that anterior nasal volume, inspiratory and expiratory nasal airway resistance (NAR) on rhinomanometry assessment were sensitive measurements to assess for severe deviation in the anterior nasal cavity. However, these measurements were less sensitive for middle and posterior septal deviation, limiting their role in assessing patients with middle or posterior septal deviation.^22^ In this study, anterior deviation was defined as septal deviation less than 2.5 cm from the middle of columella, middle septal deviation between 2.5 cm and 4.5 cm from the middle of columella and posterior deviation between 4.5 cm and 8 cm from the middle columella. Septal deviation was classified as severe if the deviation occluded more than 50 per cent of the nasal cavity and moderate if it occluded less than 50 per cent of the nasal cavity on clinical examination.

Other evidence in the literature suggests a limited role for rhinomanometry in assessing nasal airway patency^26^^–^^30^ (Table 3). Panagou *et al*. reported a moderate correlation between modified rhinomanometry (i.e., occlusion method) and subjective clinician assessment of nasal airflow on a six-point scale.^26^ In this study, each nostril was rated on a three-point scale (0 = no obstruction, 1= significant reduction of airflow compared to the contralateral side and 2 = nearly complete or complete obstruction of nasal airflow). The sum of score of each nostril provided the subjective clinical assessment score for nasal obstruction. Tompos *et al*. highlighted a very weak correlation between rhinoscopy findings and nasal airflow resistance on rhinomanometry.^28^ Huygen *et al*. reported abnormally low flow rates on rhinospirometry assessment for patients with no septal deviation or mild septal deviation restricted to one anatomical area, limiting the role of rhinomanometric evaluation in selecting patients for septoplasty. The mean detection rates of clinically diagnosed small, moderate and large septal deviations using rhinomanometry were 22 per cent, 36 per cent and 76 per cent, respectively.^29^

Pirila *et al*. highlighted that the pre-operative post-decongestant inter-cavital air flow ratio (i.e., the ratio between the flow on the deviated side and the wide side of the nasal cavity) is a strong predictor for post-operative satisfaction. With anterior rhinoscopy, the optimal cutoff value to predict post-operative satisfaction was between severe and moderate septal deviation. Pirila *et al*. concluded that, for patients with very severe septal deviation, anterior rhinoscopy was predictive of post-operative satisfaction but for patients with milder septal deviation, rhinomanometry was a useful tool for predicting post-operative satisfaction when compared to anterior rhinoscopy.^25^ Similarly, Sipilä *et al*. and Suonpää *et al*. also highlighted those patients with a deviated septum had a higher pre-operative nasal airway resistance on rhinomanometry when compared to the control cohort and pre-operative rhinomanometry assessment was predictive of post-operative satisfaction^31^^,^^32^ (Table 3).

### Relationship between clinical examination findings and PNIF

Evidence in the literature evaluating the relationship between clinical examination findings and PNIF is limited and is all level 4 cohort studies/case series (Table 4). Panagou *et al*. highlighted that the use of PNIF as an index of nasal patency measure is limited when compared to clinical evaluation of nasal obstruction.^26^ In this study, the severity of nasal obstruction was evaluated subjectively by clinicians estimating nasal flow airflow using a six-point scale. Similarly, Rujanavej *et al*. highlighted that the correlation between PNIF measurements and sinonasal disease identified on nasal endoscopy was very weak (Table 4). In this study, PNIF at a cutoff value of 90 L/min showed high sensitivity but a low specificity when compared to anterior rhinomanometry. The high sensitivity of PNIF at a cutoff value of 90 L/min may aid clinicians in identifying patients with sinonasal disease but its low specificity may result in difficulty in interpreting PNIF values in healthy asymptomatic individuals without sinonasal disease.^33^

**Table 4.** Studies investigating the correlation between clinical examination findings and PNIF

Study	Country	Evidence type/level	No. of participants (n)	Objective nasal patency measure	Summary of findings
Panagou *et al*. (2009)^26^	Greece	Cohort study/level 4	254	PNIF	The use of PNIF as indices for nasal obstruction is limited when compared to clinical evaluation of nasal obstruction on a six-point scale.
Rujanavej *et al*. (2012)^33^	Thailand	Cohort study/level 4	141	PNIF	PNIF cutoff of value of 90 L/min was considered “normal” for patients without sinonasal disease. PNIF cutoff value of 90 L/min showed a high sensitivity but a low specificity and the agreement between PNIF measurements and sinonasal disease on nasoendoscopy was only 0.09 (−0.083−0.265).

### Relationship between clinical examination findings and rhinospirometry

Studies exploring the relationship between clinical examination findings and rhinospirometry are shown in Table 5. Fyrmpas *et al*. reported that in patients with nasal partitioning ratio within normal limits (NPR value between +0.30 to -0.34 is the 95% reference range in the normal population), the agreement between nasal partitioning ratio and side of septal deviation using anterior rhinoscopy and nasal endoscopy is slight.^19^ However, in patients with a nasal partitioning ratio outside the normal range, the agreement between nasal partitioning ratio and side of septal deviation on clinical examination is substantial.^19^ Boyce *et al.* highlighted that the correlation between clinical assessment of septal deviation and nasal partitioning ratio is very strong.^34^ Clinicians are able to identify severe deviation more accurately with high sensitivity, but for patients with less severe septal deviation, the specificity of clinical examination is low^34^ and clinical examination alone may not be an alternative to rhinomanometry (Table 5).

**Table 5.** Studies investigating the correlation between clinical examination findings and rhinospirometry

Study	Country	Evidence type/level	No. of participants (n)	Objective nasal patency measure	Summary of findings
Fyrampas *et al*. (2011)^19^	Greece	Case-control study/level 4	30	Rhinospirometry	The agreement between nasal partitioning ratio and side of septal deviation was substantial for patients with nasal partitioning ratio out with normal values (k = 0.71). The agreement between nasal partitioning ratio and side of septal deviation is slight for individuals with nasal partitioning ratio within normal limits (k = 0.05).
Boyce *et al*. (2006)^34^	United Kingdom	Cohort study/level 4	46	Rhinospirometer	The correlation between clinical assessment of septal deviation and nasal partitioning ratio is very strong (correlation coefficient of 0.87). Clinical assessment had a sensitivity of 100 per cent but a specificity of only 30 per cent when compared to nasal partitioning ratio assessment.

## Discussion

### Summary of key findings

This review aimed to explore the role of objective nasal patency measures using acoustic rhinomanometry, PNIF and rhinomanometry in supporting clinical nasal examination findings in structural nasal deformities. The body of evidence evaluating the relationship between objective nasal patency measures and clinical nasal examination in the literature is small and limited to cohort studies. Several studies have suggested that nasal constrictions measured on acoustic rhinomanometry correlated well with anatomical nasal narrowing identified on nasal endoscopy and individuals with nasal obstruction generally had a lower minimal cross-sectional area on acoustic rhinomanometry when compared to healthy individuals.^11^^,^^21^^–^^23^ Acoustic rhinometry is a useful screening assessment prior to septoplasty, and this may guide clinicians to select patients who may achieve high post-operative satisfaction.^25^ However, other studies suggested that acoustic rhinometry failed to provide an exact point-to-point correlation with the nasal cavity and minimal cross-sectional areas and nasal volumes are not related to nasal obstruction secondary to septal deviation.^24^^,^^35^ Although acoustic rhinometry is useful in predicting the major sites of anatomical nasal narrowing and confirming clinical examination findings, external factors such as nasal cycle, age, posture, temperature and inter-rater variability affect objective airway testing when using acoustic rhinometry. Therefore, acoustic rhinometry may be a useful tool when used in conjunction with clinical examination findings to facilitate pre-operative planning and predicting post-operative outcomes in septoplasty. However, the use of acoustic rhinometry assessment in isolation without clinical examination may be limited by its failure to provide exact point-to-point correlation with the nasal cavity.

There is limited evidence in the literature to assess the relationship between PNIF and clinical examination findings. Current evidence suggests that the relationship between PNIF assessment and clinical examination findings is very weak.^26^^,^^33^ A cutoff value of 90 L/min on PNIF was considered “normal” for patients without sinonasal disease.^26^ However, this cutoff value has a high sensitivity but a low specificity when compared to anterior rhinomanometry, making clinical interpretation of PNIF values difficult in patients with nasal obstruction. The high sensitivity of PNIF may aid clinicians in identifying patients with sinonasal disease but its low specificity may result in difficulty in interpreting low PNIF values in healthy asymptomatic individuals without sinonasal disease. The bilateral nature of PNIF assessment and the fact that the nasal valve and alar collapse during rapid inspiration causing some degree of airway block make PNIF less reliable when assessing nasal blockage. The use of PNIF as a screening tool for nasal obstruction has not been widely explored and further research focusing on unilateral PNIF may be useful in the future to assess patients with varying degrees of nasal obstruction.

Rhinomanometry assessment correlates well with clinical examination findings in severe anterior septal deviation.^22^ However, the role of rhinospirometry in assessing middle/posterior and mild/moderate septal deviation remains uncertain. Rhinomanometry assessment should be interpreted with caution as abnormally low flow rates on rhinomanometry have been previously detected in patients in patients with no septal deviation or mild deviation restricted to one anatomical area.^29^ Rhinomanometry is also a useful screening assessment tool prior to septoplasty to guide clinicians in selecting patients who may achieve high post-operative satisfaction.^25^^,^^31^^,^^32^ Pirila *et al*. suggested that the pre-operative post-decongestant inter-cavital airflow ratio is a strong predictor for post-operative satisfaction and the optimum suggested cut of value is 1:2.^25^ The sensitivity and specificity of this cutoff value were 65 per cent and 60 per cent respectively for patients reporting high and very high post-operative satisfaction when compared to the sensitivity/specificity of anterior rhinoscopy (55%/55%). Although the sensitivity/specificity of this measurement is higher when compared to anterior rhinoscopy, the results should be interpreted with caution given the difference in sensitivity and specificity between rhinomanometry measurement and anterior rhinoscopy in assessing post-operative satisfaction is marginal.

The relationship between clinical examination findings and rhinospirometry also remains inconclusive. Nasal partitioning ratio measured from rhinospirometry is highly predictive of severe septal deviation. Although clinicians were able to differentiate severe septal deviation from moderate/mild deviation on clinical examination easily, differentiating less severe deviation from severe remains a challenge for clinicians. The sensitivity of clinical assessment of septal deviation is high but its specificity is low when compared to nasal partitioning ratio assessment in rhinospirometry. Although clinicians are able to identify severe septal deviations with NPR outside the normal range, the ability of clinicians to confidently differentiate less severe septal deviation from normal and abnormal NPR remains poor. Hence, nasal partitioning ratio assessment in rhinospirometry may be useful in assessing less severe septal deviation when used in conjunction with clinical examination findings.^19^^,^^34^

- The role of objective nasal patency measures (ONPM) in assessing structural nasal obstruction remains uncertain.
- Studies exploring the relationship between ONPM and clinical nasal examination findings are limited and confined to cohort studies in the literature.
- Acoustic rhinometry, rhinomanometry and rhinospirometry assessment correlate positively in severe anterior septal deviation but their role in assessing middle/posterior and mild/moderate septal deviation in isolation remains uncertain.
- ONPM supports clinical examination findings in severe structural nasal obstruction. Using ONPM alongside clinical examination findings may aid patient selection for septoplasty and predict post-operative satisfaction.

### Risk of bias and limitations

Most of the evidence described in this review was derived from low-level findings from cohort studies and case series. The participants from these cohort studies were heterogeneous and subjected to selection, publication and ascertainment bias. A meta-analysis was also not possible due to limited and heterogeneous evidence in the literature. The focus of the published literature has mainly been on septal deviation and other causes of nasal obstruction secondary to structural sinonasal disease such as nasal polyps and trauma remain unclear. Non-English primary articles and studies involving children were also excluded in this review.

### Implication for research and clinical practice

Evidence from this review highlights that objective nasal patency measuring tools have a limited role in supporting clinical examination findings. Although objective nasal patency measures are useful in identifying patients with severe nasal obstruction, their utility in investigating mild or moderate nasal obstruction remains poor. Objective nasal patency measures can be used in conjunction with clinical examination findings to aid patient selection for septoplasty and predict post-operative satisfaction. Further research in this field is necessary to investigate how objective nasal patency measures can be used as an objective diagnostic tool when assessing severe, moderate and mild nasal obstruction.

## Data Availability

The data that support the findings of this study are available from the corresponding author upon reasonable request.

## Appendix 1

The literature search was performed and reviewed by two independent assessors (PM and IL) using the following databases: Pubmed (US National Library of Medicine), Medline, Embase, SCOPUS, Web of Science, Cumulated Index to Nursing and Allied Health Literature (CINAHL), Cochrane Library and Google Scholar.

## Medline/Web of science

((Video endoscop* or nasal endoscop* or clinical examination* or anterior rhinoscope*) and (rhinometry, acoustic or acoustic rhinometry or PNIF or peak nasal inspiratory flow or rhinospirometer or rhinomanometer or nasal patency or nasal partitioning ratio))

## Pubmed/Embase/CINAHL/SCOPUS/COCHRANE

((“Video endoscop*” or “nasal endoscop*” or “clinical examination*” or “anterior rhinoscope*”) and (“rhinometry, acoustic” or “acoustic rhinometry” or “PNIF” or “peak nasal inspiratory flow” or “rhinospirometer” or “rhinomanometer” or “nasal patency” or “nasal partitioning ratio”))

A random search was also performed on Google scholar and Google using the search terms above. Additional articles identified from citation lists of articles searched from the databases mentioned above were also supplemented during the review stage. Articles exploring the relationship between clinical examination and objective nasal patency measures were included in the synthesis of results. The titles and abstracts were screened independently by reviewers (PM and IL) as per the inclusion and exclusion criteria outlined in Table 1. In the event of uncertainties in including or excluding an article following the assessment of the inclusion criteria, a discussion was held between the reviewers (PM and IL) to reach a consensus. All articles included following title and abstract screening were reviewed in full for further inclusion/exclusion. A narrative synthesis of the articles included was provided to explore the relationship between clinical examination findings and objective nasal patency measures.
